# Small intestinal obstruction due to phytobezoar: a case report

**DOI:** 10.1186/1752-1947-3-9312

**Published:** 2009-12-02

**Authors:** Rajan Fuad Ezzat, Shahzad Ali Rashid, Abbas Tahir Rashid, Khaled Musttafa Abdullah, Shyaw Mahmood Ahmed

**Affiliations:** 1 Department of Surgery, Sulaimanyah Teaching Hospital, Sulaimanyah, Iraq

## Abstract

**Introduction:**

Patients with mechanical small-bowel obstructions usually present with abdominal pain, vomiting, absolute constipation and varying degrees of abdominal distention. Causes can be classified as benign or malignant, or as extra- or intraluminal. A bezoar occurs most commonly in patients with impaired gastrointestinal motility. In edentulous older patients with abnormal food habits, it can also be an intestinal concretion that fails to pass along the alimentary canal.

Small bowel phytobezoars are rare and almost always obstructive. In a normal stomach, vegetable fibres that cannot pass through the pylorus undergo hydrolysis within the stomach, which softens them enough to go through the small bowel.

We present an unusual case of small intestinal obstruction caused by a phytobezoar in a patient who had neither a history of gastric surgery nor of intestinal pathology.

**Case presentation:**

A 70-year-old Iraqi Kurdish man was hospitalized due to abdominal pain, vomiting and dehydration. Investigations concluded small intestinal obstruction. Subsequent laparotomy revealed that the cause of the obstruction was an eggplant phytobezoar.

**Conclusion:**

Many types of bezoar can be removed endoscopically, but some will require operative intervention. Subsequently, prevention of any recurrence should be emphasized.

## Introduction

Phytobezoars are a concretion of poorly digested fruit and vegetable fibres that are found in the alimentary tract. These usually take the form of orange pith or pulp in patients with a history of surgery, or persimmon in patients without previous surgery [1].

Persimmon contains a high concentration of tannin, a monomer that polymerizes in the presence of gastric acid. The polymerized tannin then acts as a nucleus for bezoar formation. In a normal stomach, vegetable fibres that cannot pass through the pylorus undergo hydrolysis within the stomach, which softens them enough to go through the small bowel. In patients who have undergone gastric surgery, however, gastric motility is disturbed and gastric acidity is decreased, and the stomach may empty rapidly with an increased possibility of bezoar formation.

Normally found in the stomach, bezoars may pass through the small bowel. Primary small bowel bezoar is very rare and normally forms in patients with an underlying small bowel disease like diverticulum, stricture or tumour. Phytobezoar can also develop secondarily if there are areas of sufficient stagnation within a dilated bowel segment. This may occur in patients with strictures caused by Crohn's disease, tuberculosis or previous surgery, or in patients with small bowel diverticula. In such cases, bile constituents or calcium salts contribute to bezoar development [3]. We present an unusual case of small intestinal obstruction caused by phytobezoar although the patient had neither a history of gastric surgery nor of intestinal pathology.

In this case, swallowed foreign bodies may have been involved although a foreign body that has passed the pylorus is usually able to pass through the remainder of the small bowel without difficulty, unless the small bowel is already compromised by postoperative adhesions.

The terminal ileum is the narrowest part of the small bowel, and peristalsis may be weaker here than in more proximal segments. The intramural width of the small bowel may be measured by taking plain abdominal radiographs of a gas-filled lumen. An intramural width of 3 cm is considered abnormal and may indicate obstruction or ileus.

Certain radiologic investigations can be used to confirm the diagnosis and severity of a small-bowel obstruction, but not its etiology. Others are aimed at determining the cause of small-bowel obstructions [4]. Conventional plain radiography is the investigation of choice for patients with suspected small-bowel obstructions, and this method should always be performed first [4].

A bowel larger than 3 cm in diameter is often associated with obstruction. Gas and fluid is usually present in the distended small bowel loops, and gas and fluid levels may be present at the same or different levels in the abdominal cavity [4].

## Case presentation

A 70-year-old Iraqi Kurdish man was referred to our centre for further management of intestinal obstruction. He presented with a history of a few hours of epigastric discomfort associated with vomiting and abdominal distension. His bowel habit was mildly altered but there was no history of rectally passing blood. He denied any loss of weight or appetite. Medically he was being treated for hypertension and congestive cardiac failure. His past surgical history consisted of cardiac catherization and angiography 1 year before presentation.

**Figure 1:**
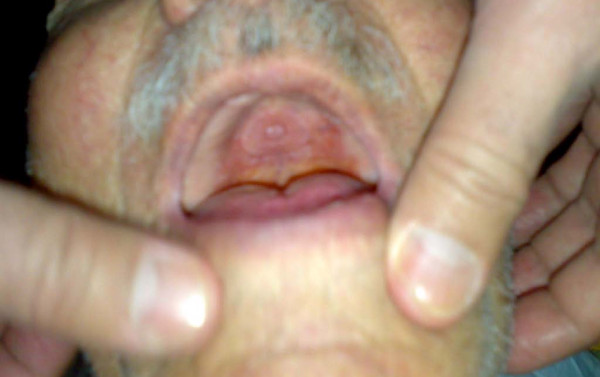
**The edentulous patient**.

His vital signs upon admission were stable with blood pressure at 140/90 mmHg and a heart rate of 100 beats/minute. His abdomen was tender but slightly distended. Bowel sound was sluggish and rectal examination revealed an empty rectum with no palpable mass. His hernia orifices were normal and he was also edentulous (Figure 1).

**Figure 2:**
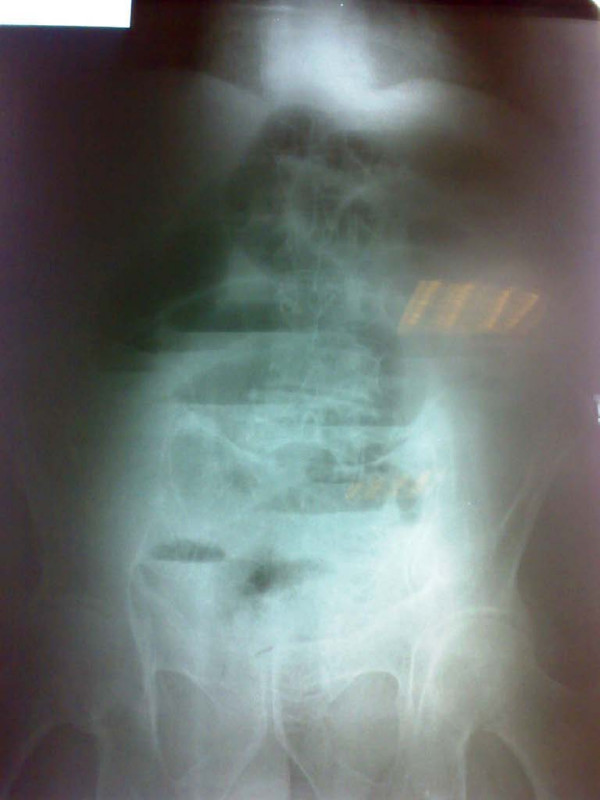
**Small intestinal obstruction**.

**Figure 3:**
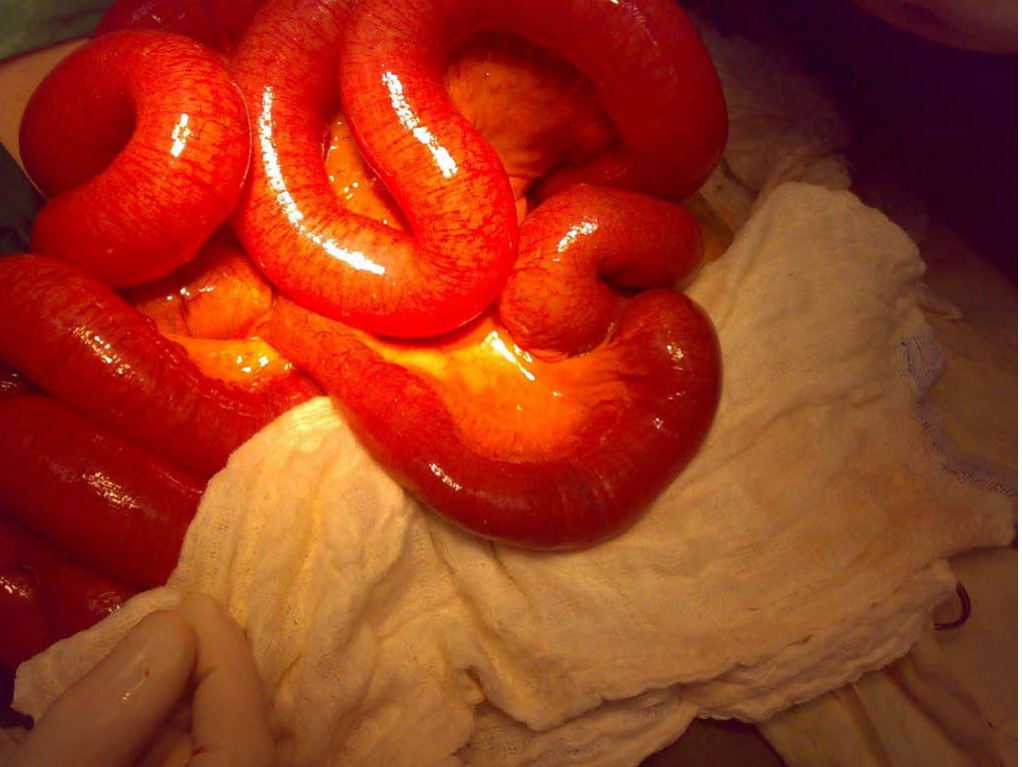
**Hard object in terminal ileum with small intestinal obstruction**.

**Figure 4:**
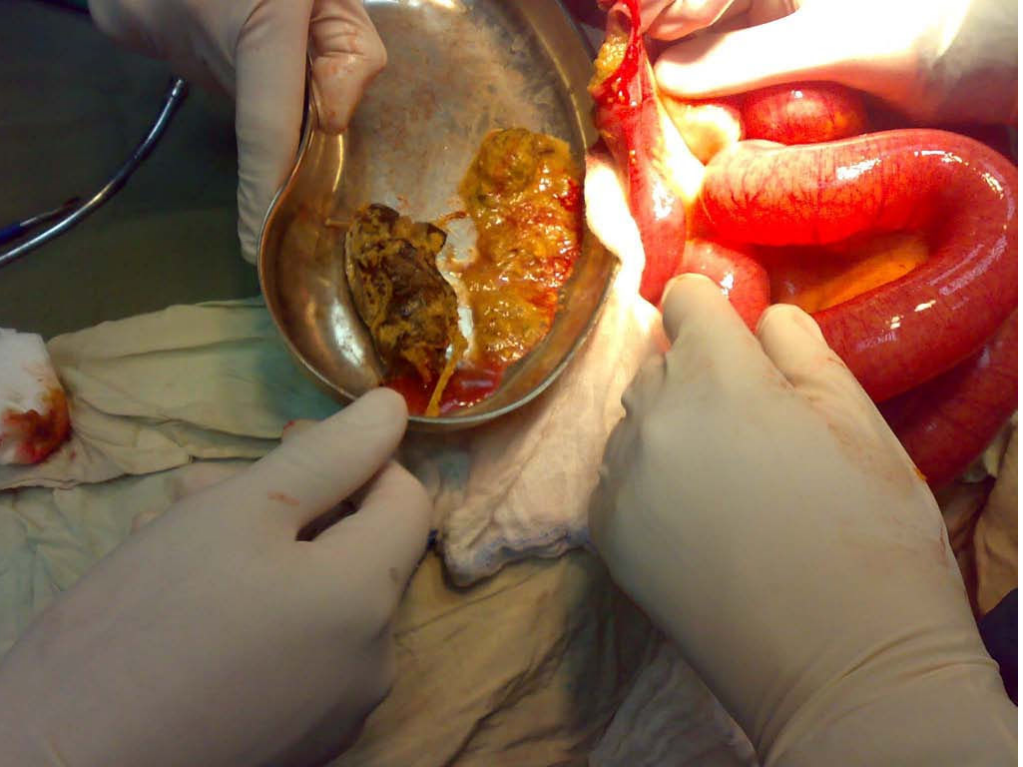
**Ileotomy for extraction of the eggplant**.

**Figure 5:**
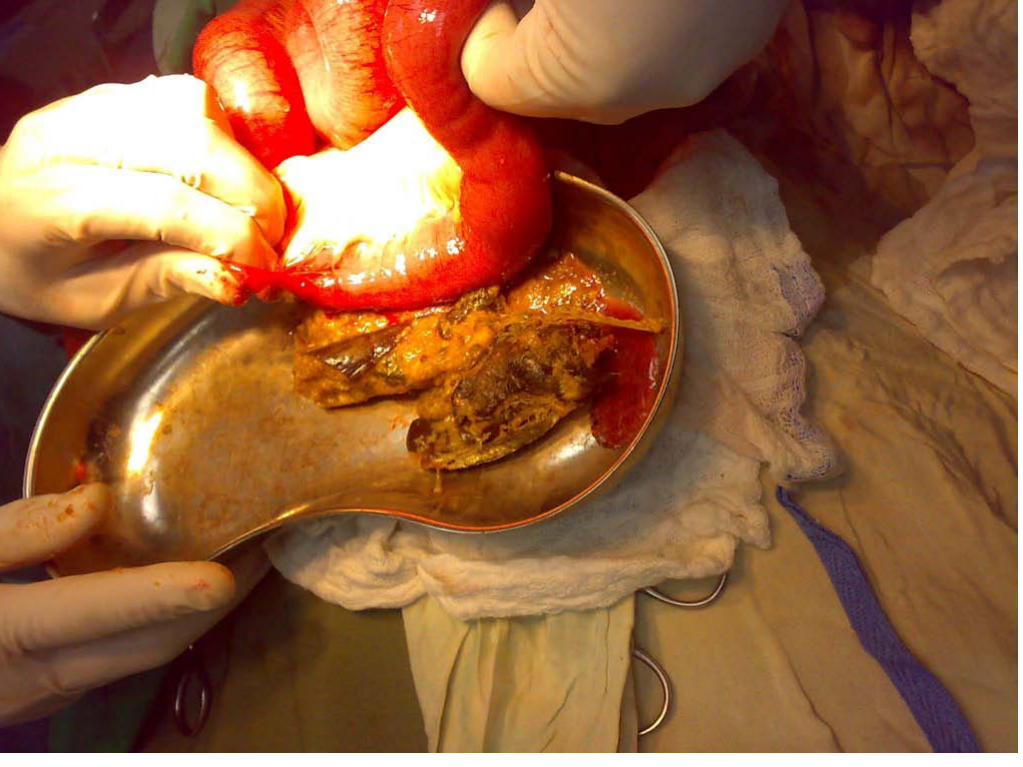
**Eggplant in kidney dish**.

His electocardiogram showed evidence of old ischemic changes. His blood investigation results were unremarkable. A clinical diagnosis of intestinal obstruction was then made based on his radiological findings (Figure 2). An exploratory laparotomy was subsequently performed on the patient, which yielded findings of a hard intraluminal body obstructing the terminal ileum (Figure 3). The operation confirmed suspicion of a bezoar measuring 5 × 3 cm, which was found at a distance of 10 cm from the ileocaecal junction (Figure 4) exteriorized through ileotomy (Figure 4 and 5). His jejunum and ileum were dilated and hypertrophied but no jejunal or ileal mass or polyps were found.

The pathology report of the operative specimen was degenerate vegetable matter. The postoperative period was uneventful, during which the patient was started on nourishing fluid and a soft diet. He was discharged 4 days later. After 1 week he was found to be well during follow-up in surgical clinic.

## Discussion

Small bowel obstructions account for 20% of hospital admissions. Common causes are adhesions, strangulated hernia, malignancy, volvulus and inflammatory bowel disease. Phytobezoars are rare, accounting for only 0.4 to 4% of all cases of intestinal obstruction. No particular age or sex prevalence has been observed [5].

There are four types of bezoars - phytobezoars, trichobezoars, pharmacobezoars and lactobezoars. Phytobezoars are the most common, and are composed of vegetable matter (celery, pumpkin, grape skin, prune and persimmons) and contain a large amount of non-digestible fibres (cellulose, hemicellulose, lignin and fruit tannins). On the other hand, trichobezoars are gastric concretion of hair fibres which usually presents in patients with a history of psychiatric predisposition and in children with mental retardation. Meanwhile, pharmacobezoars consist of medication bezoars, such as cholestyramine, kayexalate resin, cavafate and antacids, which adhere when in bulk. Lastly, lactobezoars are milk curd secondary to infant formula, described in low birth weight neonates fed on highly concentrated formula within their first week of life [6].

Primary small bowel bezoars almost always present as intestinal obstructions. They usually become impacted in the narrowest portion of the small bowel, the most common site being the terminal ileum, as was found in our patient, followed by the jejunum [8]. It is interesting to note that more than half of reported cases of patients with phytobezoars had a history of gastric surgery [8]. Our patient gave no history of gastrointestinal-related surgery.

A plain radiograph typically shows a classic obstructive pattern. Occasionally we may be able to see the outline of a bezoar, which is actually difficult to differentiate from abscess or feces within the colon. Ultrasound has been used to detect bezoar. In a retrospective study done by Ripolles *et al*. [9], ultrasound was able to detect phytobezoar in 88% of patients with small bowel obstructions. A bezoar appears as a hyperechoic arc-like surface with acoustic shadowing on ultrasound; however this feature may cause difficulty in differentiating bezoar from gallstones, which have similar ultrasound characteristics.

## Conclusion

We present an uncommon case of small bowel obstruction caused by a secondary phytobezoar that passed the pylorus without digestion.

Small bowel bezoars are treated surgically. It is mandatory to explore the whole gastrointestinal tract in order to avoid synchronous bezoar and the recurrence of intestinal obstruction due to a retained bezoar. Other treatment options include enzymatic breakdown and endoscopic fragmentation for a gastric bezoar [1,5].

Recurrence is common unless the underlying predisposing condition is corrected. Prevention includes avoidance of high-fibre foods, introduction of prophylactic medication to improve gastric emptying and psychological or psychiatric follow-up in patients with psychiatric disease [5]. In difficult, recurrent cases, periodic endoscopy with repeated mechanical disruption is necessary.

## Consent

Written informed consent was obtained from the patient for publication of this case report and any accompanying images. A copy of the written consent is available for review by the Editor-in-Chief of this journal.

## Competing interests

The authors declare that they have no competing interests.

## Authors' contributions

RE analyzed and interpreted the patient's data, and operated on the patient. SR assisted in the operation and analysed the patient's data. AR assisted in the operation and followed up the patient. KA collected the patient's data and followed up the patient. SA admitted the patient in the casualty department and took his history.
